# Olfactory impairment in Italian patients with chronic rhinosinusitis with nasal polyps: a patient-centered survey

**DOI:** 10.3389/falgy.2024.1519069

**Published:** 2025-01-07

**Authors:** Francesca Anastasi, Frank Rikki Mauritz Canevari, Stefania Gallo, Giulia Gramellini, Enrico Heffler, Ignazio La Mantia, Giulia Monti, Martina Ragusa, Alberto Macchi

**Affiliations:** ^1^ Unit of Otorhinolaryngology, Medical and Surgical Rhinology, Ospedale San Giovanni Evangelista, Tivoli, Italy; ^2^ Facoltà Dipartimentale di Medicina, UniCamillus—Saint Camillus International University of Health and Medical Sciences, Rome, Italy; ^3^ Unit of Otorhinolaryngology-Head and Neck Surgery, IRCCS Ospedale Policlinico San Martino Genoa, Genoa, Italy; ^4^ Department of Surgical Sciences Integrated Diagnostics (DISC), University of Genoa, Genoa, Italy; ^5^ Unit of Otorhinolaryngology, Head and Neck Department, ASST Sette Laghi, Varese and UPLOAD (Upper and Lower Airways Diseases) Research Centre, University of Insubria, Varese, Italy; ^6^ Unit of Otorhinolaryngology, ASST GOM Niguarda, Milan, Italy; ^7^ Department of Biomedical Sciences, Humanitas University, Pieve Emanuele, Milan, Italy; ^8^ Personalized Medicine, Asthma and Allergy, IRCCS Humanitas Research Hospital, Rozzano, Milan, Italy; ^9^ Unit of Otorhinolaryngology, Department of Medical, Surgical, and Advanced Technologies “G.F. Ingrassia”, University of Catania, Catania, Italy; ^10^ Unit of Otorhinolaryngology-Head and Neck Department, ASST Sette Laghi, University of Insubria, Varese, Italy; ^11^ Department of Medical-Surgical Sciences and Advanced Technologies, E.N.T. Unit, P.O. “G. Rodolico”, University of Catania, Catania, Italy; ^12^ Unit of Otorhinolaryngology-Head and Neck Department, ASST Sette Laghi, Varese and UPLOAD (Upper and Lower Airways Diseases) Research Centre, University of Insubria, Varese, Italy

**Keywords:** biologic therapy, chronic rhinosinusitis with nasal polyps (CRSwNP), dupilumab, olfactory restoration, smell

## Abstract

**Background:**

Chronic rhinosinusitis with nasal polyps (CRSwNP) is an inflammatory condition characterized by persistent nasal obstruction, discharge, facial pressure, and olfactory dysfunction. CRSwNP significantly impairs quality of life (QoL), with olfactory loss being a particularly distressing symptom that affects food enjoyment, personal safety, and social interactions.

**Methods:**

This study investigated the experiences of Italian patients with CRSwNP. A cross-sectional quantitative and qualitative survey (Google Forms questionnaire), collecting data on diagnosis, symptomatology, QoL impact, and treatment experiences was developed and distributed.

**Results:**

There were 155 respondents, with over half diagnosed with CRSwNP for more than a decade. Nasal obstruction was the primary symptom leading to medical consultation. Corticosteroid therapy and surgery showed limited and variable effectiveness in olfactory restoration. Biologic therapy, particularly dupilumab, demonstrated promising results, with approximately half of the patients reporting complete smell restoration.

**Conclusions:**

CRSwNP significantly affected patients' QoL, with olfactory dysfunction being a common and impactful symptom. While current treatments provide symptom relief, they do not always result in sustained olfactory improvement. Biologic therapy emerged as a promising option for olfactory restoration, underscoring the importance of personalized treatment strategies. Further research is warranted to explore the mechanisms of olfactory recovery and to optimize treatment protocols for CRSwNP.

## 1 Introduction

Chronic rhinosinusitis (CRS) is a common inflammatory condition of the nasal cavities and sinuses, defined by two or more symptoms persisting for more than 12 weeks (1). It is traditionally classified into CRS with nasal polyps (CRSwNP) and CRS without nasal polyps (CRSsNP), but this classification has limitations as it oversimplifies the disease's complexity (2). As such, the 2020 European Position Paper on Rhinosinusitis and Nasal Polyps (EPOS) proposed a new classification considering comorbidities, extent and distribution of the condition, endotype dominance, and clinical phenotypes (3).

CRSwNP affects about 25%–30% of patients with CRS (4) and varies in prevalence across regions (5–8). CRSwNP pathogenesis used to be categorized as a type 2 inflammatory response marked by inflammation of the nasal and paranasal mucosa, but not all nasal polyps exhibit these elevations; comorbidities do not by themselves define CRSwNP (4), which is diagnosed by evidence of nasal polyps or sinus opacity on radiology (4). Symptoms include nasal obstruction, nasal discharge, facial pressure, and hyposmia or anosmia (9, 10). CRSwNP significantly impacts quality of life (QoL), especially during acute exacerbations or when comorbidities such as asthma are present (3, 11, 12). It is also associated with increased depression and social dysfunction (13).

CRSwNP imposes a significant economic burden, with annual direct costs per patient estimated at USD 2609, GBP 2974, and EUR 1501 (3). Indirect costs, encompassing healthcare utilization, absenteeism, and lost work productivity, can escalate to EUR 5659 (14, 15). Management of CRSwNP is complex and often contentious, as current treatments do not offer a cure (16). The primary treatment goal is symptom control and disease impact reduction (17), employing strategies such as saline sinus rinses, corticosteroids, antibiotics (18), and adjunctive therapies (19, 20). Although not curative or restorative, surgical interventions, such as endoscopic sinus surgery, may often be required; surgery is expected to facilitate more appropriate treatment options (1). Upon careful endotyping, some patients may be candidates for biologic therapies aimed at specific inflammatory pathways, such as anti-interleukin (IL)4-receptor alpha, anti-IL5, and anti-immunoglobulin E agents (21).

Olfactory dysfunction significantly impacts QoL, making everyday activities considerably more challenging (22–24). It affects the enjoyment of food and drink, food perception, food-evoked emotions, dietary patterns, and overall life satisfaction (25–27). Furthermore, anosmia and hyposmia are associated with increased anxiety and depression symptoms, leading to a decreased QoL (28–30). The implications of olfactory dysfunction extend beyond QoL, increasing the risk of exposure to environmental hazards and food poisoning (23), thereby compromising personal protection and safety (31). It can also impact an individual's perception of the environment, including food perception and the perception of odors (25).

Patients with CRSwNP often experience poorer QoL, higher anxiety and depression symptoms, and compromised olfactory function compared with patients with allergic rhinitis (32). Olfactory impairment can lead to disruptions in bonding within close social relationships, decreased social support, and altered sexual behaviors (33). It can significantly impact certain professions and job duties, such as those dependent on olfaction for safety or livelihood (34).

Finally, a decreased sense of smell can lead to taste disturbances, and loss of pleasure from eating, resulting in weight changes and potential difficulties in avoiding health risks (35). Despite this, studies focusing on the experience of patients with CRSwNP and smell disturbances are limited. This study aims to address this gap by exploring the experiences of Italian patients living with CRSwNP and the impact of this condition on their sense of smell.

We developed and distributed a questionnaire to investigate these topics. We evaluated the effect of CRSwNP on QoL and whether biologic therapy is a suitable option for olfactory restoration.

## 2 Materials and methods

### 2.1 Study design

This study employed a cross-sectional survey design to delve into the experiences of smell in Italian patients with CRSwNP who are currently on biologic therapy.

The survey instrument was designed using Google Forms. The final draft of the survey underwent rigorous revision by the authors, which involved the input of Ear, Nose, and Throat (ENT) specialists. These specialists are actively engaged in managing patients suffering from CRSwNP across various Italian centers, thereby bringing a wealth of practical insights to the survey design.

Patients who had initiated biological therapy between 1 April 2022 and 31 March 2023 were eligible for inclusion, i.e., all patients contacted were receiving biologic therapy that had begun during the previous year. Patients were also treated with other therapies (such as oral corticosteroids) as per the recommendations of EPOS 2020 (3). Patients identified for inclusion were surveyed between March and June 2023. During this period, patients were either interviewed directly by their clinicians during routine follow-up checks or contacted via email or WhatsApp, providing them with a direct link to the Google Forms survey. This dual approach facilitated a comprehensive and flexible data collection process, accommodating the varied preferences and needs of the patients.

### 2.2 Data source and participants

The questionnaire for this study was designed to gather data on various aspects of the patient's experience with CRSwNP. It included a mix of quantitative, qualitative, open-ended, and Likert-scale questions that aimed to explore topics such as the time of diagnosis, symptoms that led the patient to seek medical help, the patient's experience with the loss of smell, their experience with corticosteroid therapy and surgical interventions, and their experience with biologic therapy.

The questionnaire also sought to understand whether the patient has experienced any unusual olfactory experiences and whether they have undergone olfactory training if they have not regained their sense of smell.

As the survey was open to any individual with a diagnosis of CRSwNP who had initiated biologic drug therapy in the year prior to participating in this survey study, many of the survey responses were based on patient recollection of symptoms prior to commencing biologic therapy. The self-reported diagnosis was then evaluated at baseline using objective and semi-objective tests, such as the Sniffin' Sticks Test (36). Patients who did not report the symptom of hyposmia at baseline were excluded from the study.

The patients involved in the survey were from second-level, university and hospital centers distributed throughout Italy, including Catania, Genoa, Milan, Varese, and Rome. The survey was free to access, and participation was voluntary.

### 2.3 Statistical analyses

For this study, only descriptive statistics were performed (using Google Forms), and no minimum sample size was required.

## 3 Results

### 3.1 Diagnosis, symptoms, and quality of life impact

The survey, completed and returned by 155 respondents, revealed a high occurrence of chronic disease, with the majority (54.2%) having been diagnosed with CRSwNP for over a decade. Nasal obstruction was the predominant symptom prompting individuals to seek medical advice, as indicated by 72.3% of the participants. Other symptoms included anosmia (17.4%), chronic nasal discharge (7.7%), and facial discomfort (2.6%).

**Figure 1:**
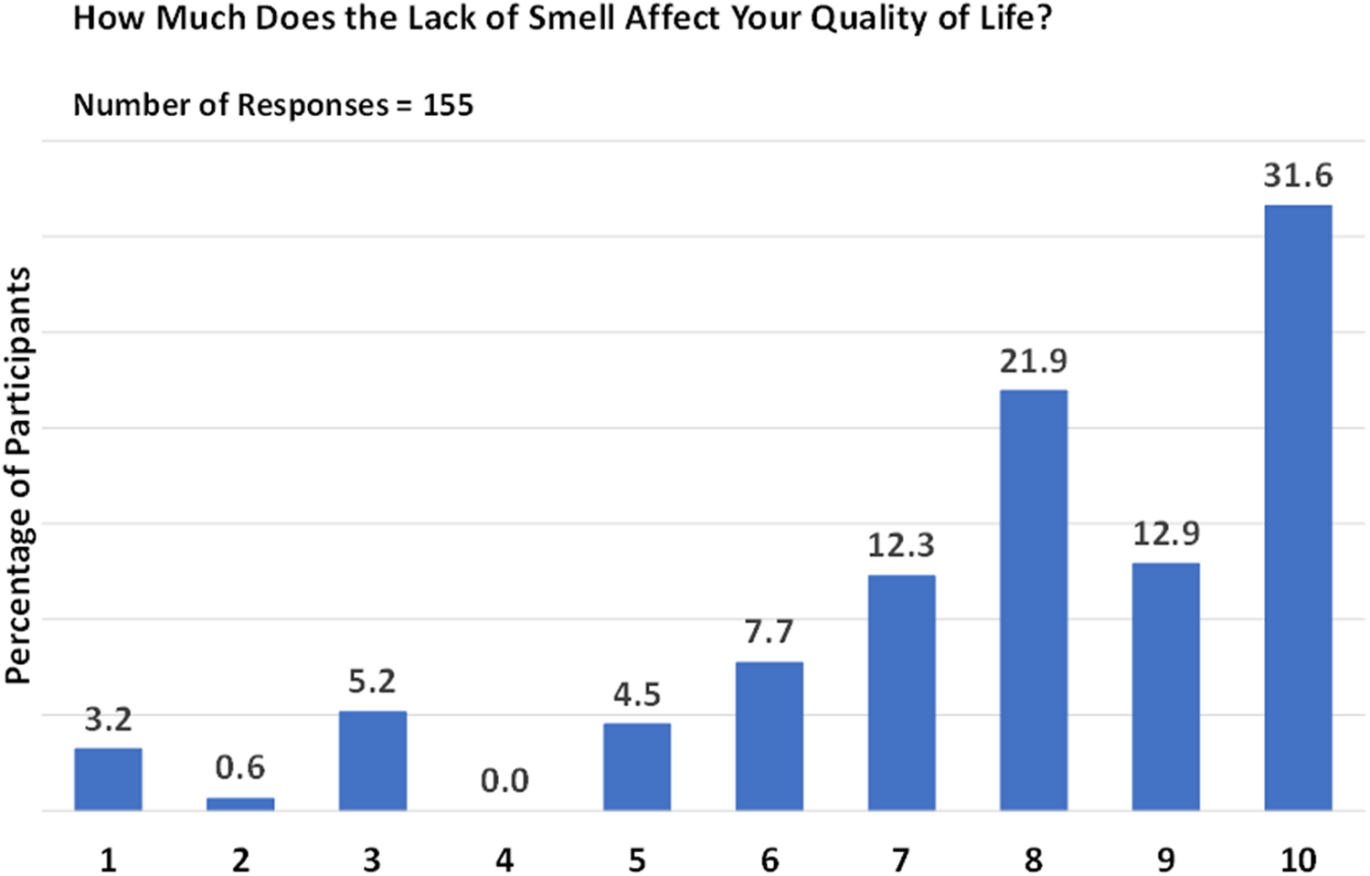
Impact of smell loss on quality of life. Responses are given on a scale from 1 (no impact) to 10 (complete life change).

Interestingly, approximately 14.2% of respondents reported a relatively recent (i.e., a few months previously) onset of anosmia, while 38.1% of patients with CRSwNP had experienced anosmia for more than ten years. The onset of smell loss was typically gradual (80.6%) rather than abrupt (19.4%), considerably impacting the QoL of 44.5% of individuals, as evidenced by ratings of 9 and 10 on a scale of 1 (no impact) to 10 (complete life change) (Figure 1). The most frequently reported rating was 10 (31.6%), followed by a rating of 8 (21.9%). Lower ratings were less common, with only a small proportion of patients assigning ratings of 1 (3.2%) or 2 (0.6%; Figure 1).

### 3.2 Oral corticosteroid therapy

**Figure 2:**
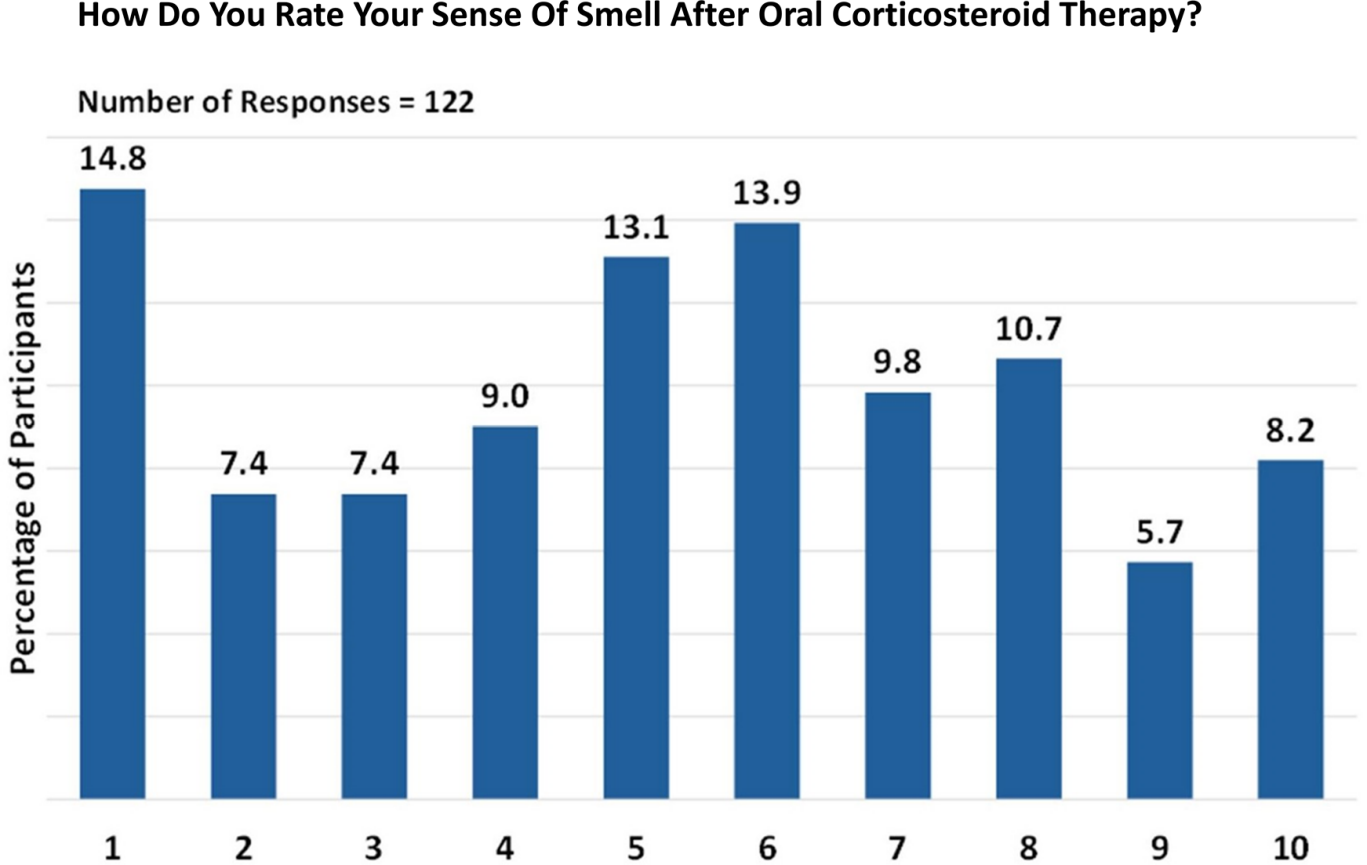
Perception of smell after oral corticosteroid therapy. Responses are given on a scale from 1 (unchanged) to 10 (fully restored).

Oral corticosteroid treatment was received by 122/155 participants for olfactory dysfunction. On a scale of 1 (unchanged) to 10 (fully restored), the most frequently observed treatment ratings were 1, 5, and 6, accounting for 14.8%, 13.1%, and 13.9% of the responses, respectively. Despite the lower ratings, the overall impact of the treatment was encouraging. A substantial proportion of the participants reported an olfaction rating of 5 or above, indicating a positive effect of oral corticosteroid treatment on their sense of smell (Figure 2).

After oral corticosteroid therapy, approximately 17.9% of patients reported no restoration of smell (0 months) while 23.6% of patients regained their sense of smell for 1 month. The remaining patients experienced varying durations of restored smell, ranging from less than 1 month (13.2%) to as long as 24 months (1.9%). While oral corticosteroid therapy can be effective in restoring the sense of smell, the duration and degree of restoration can vary significantly among patients.

### 3.3 Surgery

**Figure 3:**
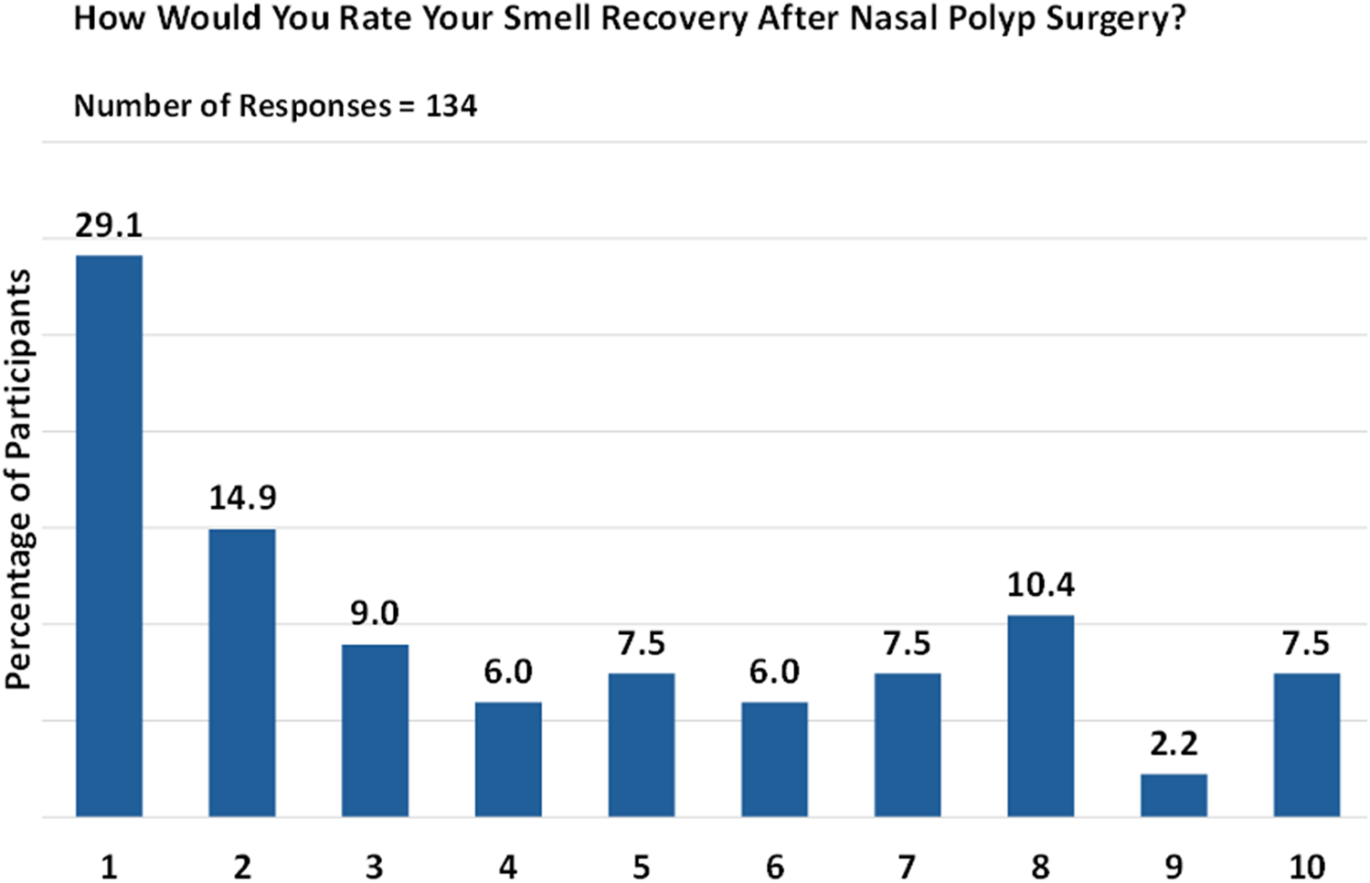
Post-surgery smell restoration. Responses are given on a scale from 1 (no smell) to 10 (complete restoration of smell).

Of the participants who underwent nasal polyp surgery and responded to the survey (*n* = 134), 44% reported low levels of smell restoration post-surgery (Figure 3). Responses were given on a scale from 1 (no smell) to 10 (complete restoration of smell). Conversely, nearly 33.6% of respondents provided positive feedback about the surgery.

For 42.6% of individuals, sense of smell either did not return or was restored for only a month or less after surgery. The duration of smell restoration varied widely, ranging from 2 to 180 months. However, long-term restoration of smell was relatively rare.

**Figure 4:**
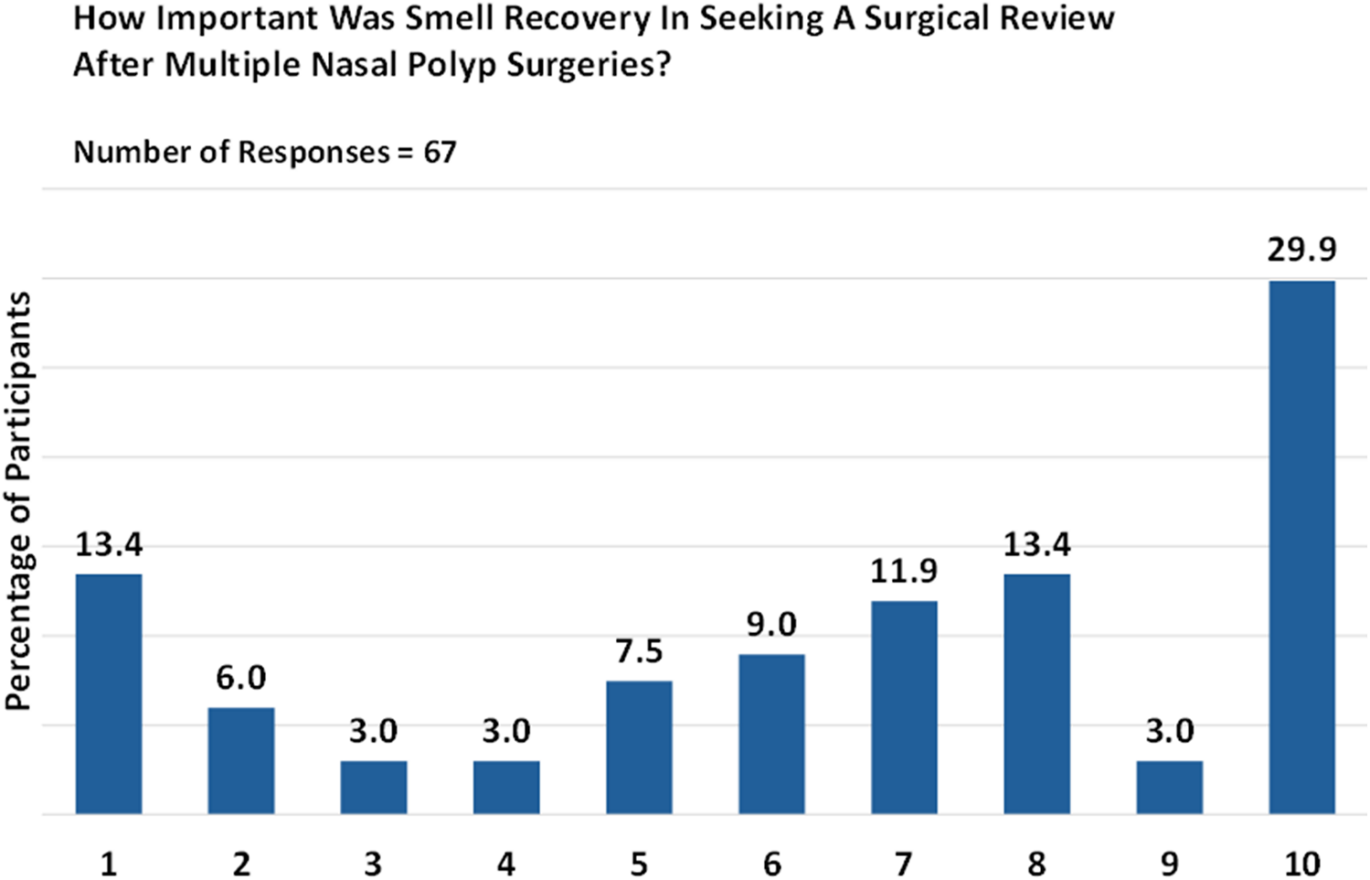
Patient priorities: the importance of smell restoration. Responses are given on a numeric scale from 1 (not at all) to 10 (critically important).

The significance of smell restoration played a pivotal role in prompting a surgical revision for just under half (44.9%) of respondents who had undergone multiple surgeries. Nevertheless, it is worth noting that smell restoration may not hold the same level of priority for all patients. Specifically, of 67 respondents, 29.9% rated smell as “critically important” (rating 10), 13.4% considered it “extremely important” (rating 8), and 11.9% found it to be “very important” (rating 7; Figure 4).

### 3.4 Biologic therapy

In our study, dupilumab was the most prescribed treatment (91.9%), followed by mepolizumab (3.4%), omalizumab (2.7%), and benralizumab (1.3%). It is important to note that the use of certain biologics, such as benralizumab, might not be directly related to the treatment of CRSwNP but could be due to other conditions like asthma.

**Figure 5:**
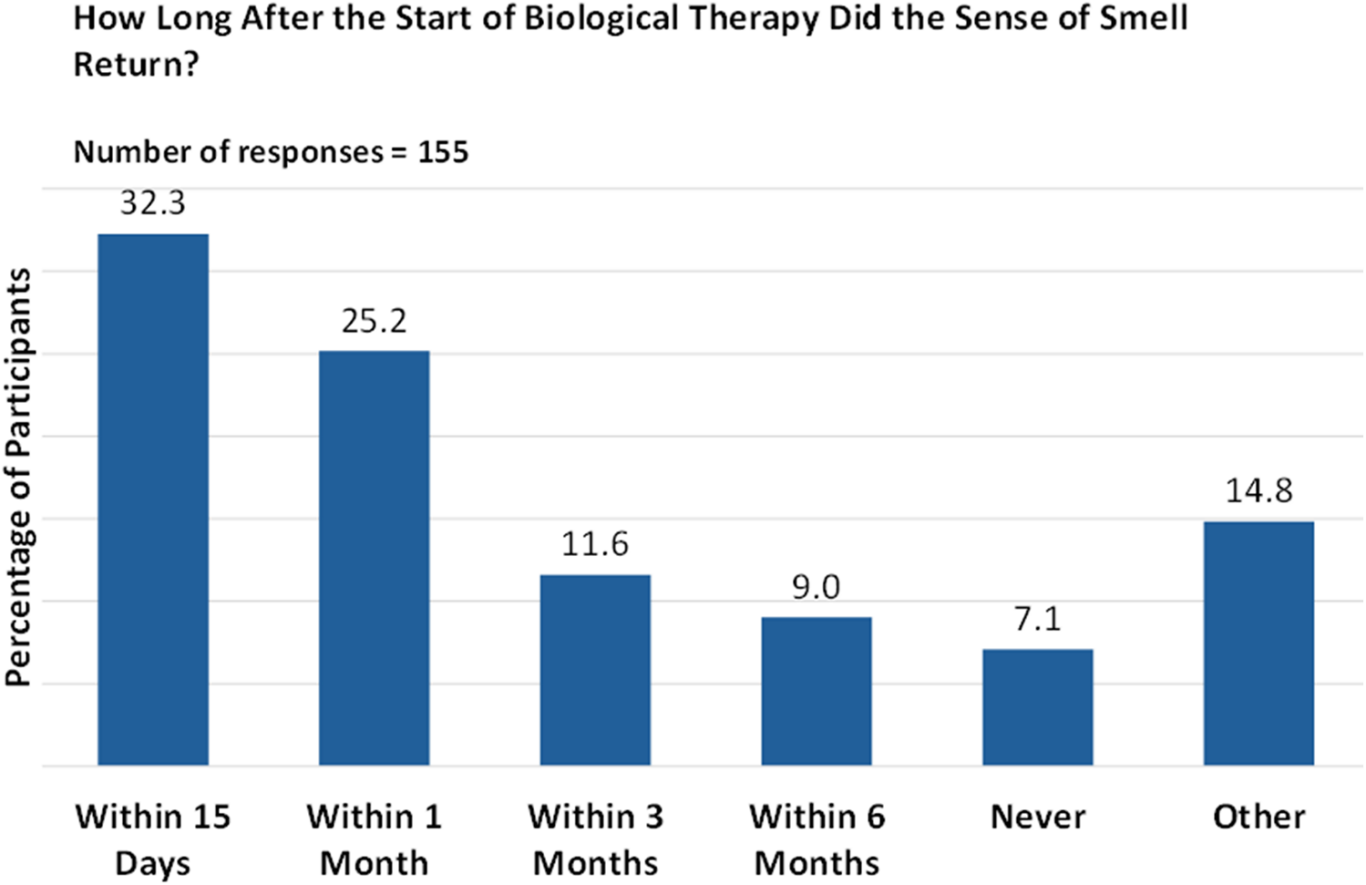
Timeframe for the return of the sense of smell after biologic therapy.

The duration of biologic therapy varied among patients, with the majority (72.4%) having been on therapy for more than six months. Notably, 32.3% of patients reported a return of their sense of smell within 15 days of starting therapy, while 25.2% experienced this within a month. However, biologic therapy was ineffective for 7.1% of respondents (Figure 5).

**Figure 6:**
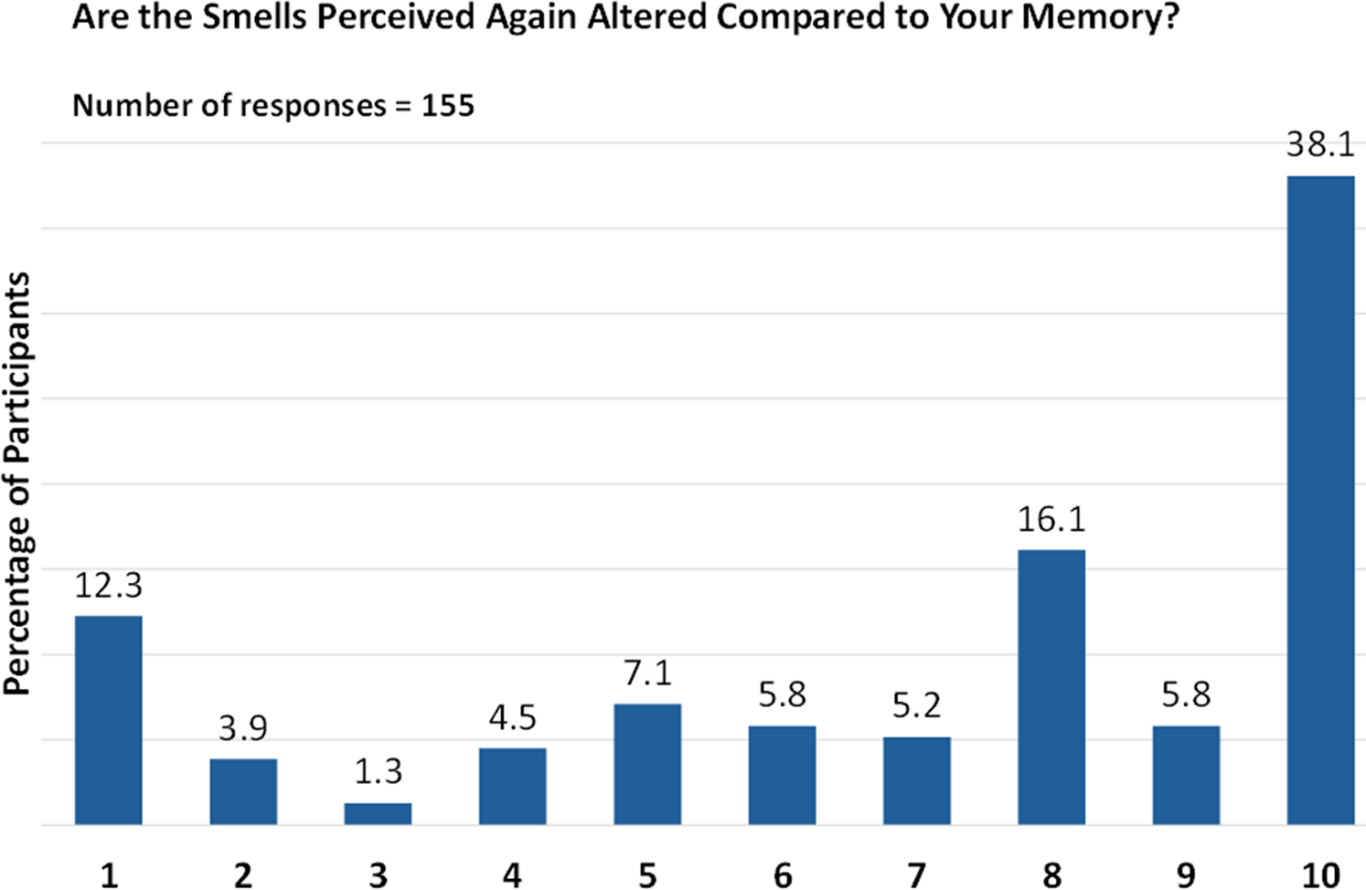
Quality of olfaction post biologic therapy. Responses are given on a scale from 1 (altered perception) to 10 (normal perception).

Interestingly, all lost smells reappeared at once for 30.3% of respondents. When asked about the quality of perceived smells, 38.1% rated it as “10” (on a scale of 1 [altered perception] to 10 [normal perception]), indicating that the smells were not altered compared with their memory (Figure 6). Cumulatively, the quality of smell perceived (on a scale of 1 to 10) was considered positive (6 to 8) or very positive (9 to 10) by 27.1% and 43.9% of respondents, respectively.

**Figure 7:**
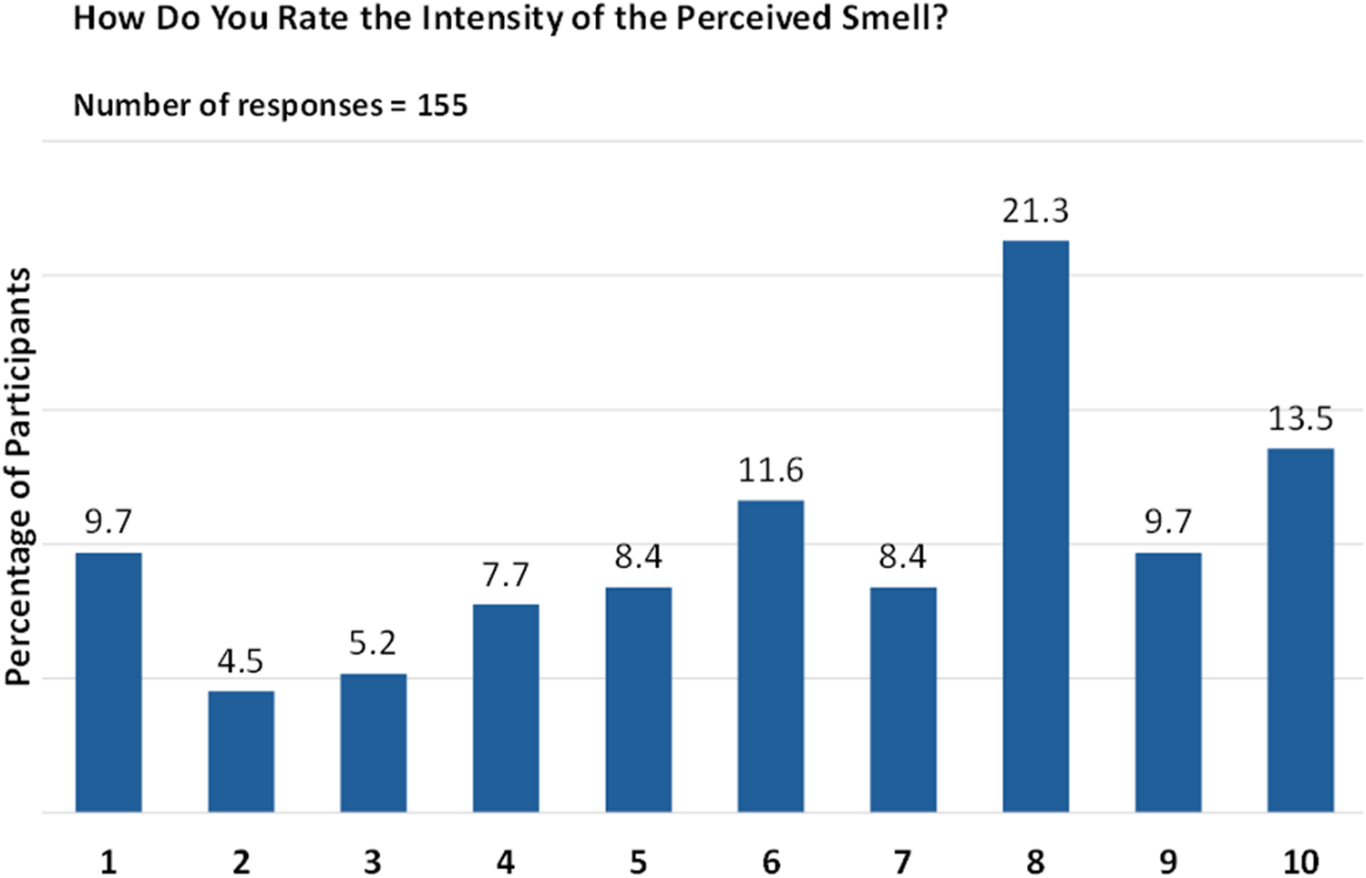
Intensity of smell perception post biologic therapy. Responses are given on a numeric scale from 1 (very faint) to 10 (very intense).

However, when rating the intensity of the perceived smell on a scale of 1 (very faint) to 10 (very intense), responses were more varied, with the highest percentage (21.3%) giving a rating of “8”, followed by 13.5% giving a rating of “10” (Figure 7).

Patients associated various sensations with the regained sense of smell, including a feeling of an unblocked nose (50.3%), lightness in breathing (31.6%), improved night rest (14.2%), and decreased snoring (3.9%). Despite these positive outcomes, only 55.5% felt they could perceive all smells after starting biologic therapy, and only 36.1% considered the restoration of smell to be the most significant change they experienced from biologic therapy. Interestingly, 35.5% reported perceiving only some smells that were lost before their restoration with biologic therapy, without any apparent explanation.

A significant portion of respondents (41.3%) reported experiencing cacosmia, or the perception of unpleasant odors before using biologic therapy. Fewer respondents (19.4%) reported experiencing parosmia, a condition where normal smells are perceived as different, often unpleasant, odors. Even fewer respondents (16.1%) reported experiencing phantosmia (the detection of smells that are not present in someone's environment). Among those who had not regained their sense of smell, only a small proportion (8.4%) reported that they had been set up for olfactory training. Among those who underwent olfactory training, a significant majority (69.6%) reported noticing improvements after using biologic therapy.

## 4 Discussion

Our investigation, involving 155 patients residing in Italy and living with CRSwNP, offers valuable insights into the impact of olfactory impairment on their lives. The survey uncovered that over 50% of patients had received their CRSwNP diagnoses over a decade ago. Olfactory dysfunction is a common symptom among patients with CRSwNP, affecting 60% to 70% of individuals, as reported in studies by Passali and colleagues (37) and Chung and colleagues (29). However, it is essential to acknowledge that this prevalence might be an overestimate. The study by Chung and colleagues included severe cases requiring surgical management of CRSwNP, which could have influenced the results (29).

Our survey indicates that while olfactory dysfunction was a chronic condition for approximately 38% of respondents, it was the primary reason for seeking medical attention in only about 17% of cases. The treatment of CRSwNP aims to alleviate symptoms and enhance patient QoL while minimizing the side effects of medications. This objective is achieved through medical management targeting the inflammation causing polyp growth and symptom development. When initial medical treatments prove ineffective, sinus surgery is considered. However, many patients with CRSwNP may require multiple treatments and potentially long-term therapy to effectively manage their symptoms.

A considerable proportion of our participants underwent corticosteroid treatment for their olfactory dysfunction. However, a significant number of patients reported no or only temporary smell restoration. This data aligns with findings from Head and colleagues who noted that three to six months after oral steroid treatment, patients showed minimal to no improvement in health-related QoL (HRQoL) or symptom severity compared with those on placebo or no treatment (38). Interestingly, the use of dupilumab in patients with CRSwNP and coexisting asthma (previously on large doses of corticosteroids) significantly improved asthma outcomes; the study also reported a concomitant reduction of both corticosteroid treatment and asthma disease burden in this patient population (39).

While surgery carries the risk of injuring olfactory epithelial surfaces and the potential for disease recurrence necessitating further surgical interventions (40, 41), it may lead to improved long-term outcomes (42, 43). Our survey shows that after surgery, 44% of patients rated the restoration of smell as low (1 or 2 on a scale of 1 to 10). However, a limitation of this survey is that the lack of data on precise types of surgery may have resulted in interpatient variability.

The duration of smell restoration varied widely (from less than a month to as long as 180 months). However, long-term restoration of smell was relatively rare. The significance of smell restoration was a key factor in prompting a surgical revision; nevertheless, it is worth noting that smell restoration may not hold the same level of priority for all patients. This highlights the need for personalized treatment approaches in managing CRSwNP.

Biologic therapy has shown promising results in managing conditions such as asthma and atopic dermatitis, leading to the exploration of monoclonal antibodies for their potential use in other areas. These antibodies, including dupilumab, omalizumab, mepolizumab, reslizumab, and benralizumab, target specific pathways involved in the development of CRS (3). Recognizing their potential, these antibodies have been repurposed to treat CRSwNP, which is driven by type 2 inflammation in over 85% of European cases (44). Biologic treatments have demonstrated remarkable and durable improvements in symptoms and clinical outcomes for patients with CRSwNP (44, 45).

All respondents to the survey had initiated biologic therapy, with dupilumab being the most prescribed treatment (about 92%). The majority had been in therapy for more than six months. Dupilumab, a newer targeted biologic therapy, has shown significant and long-lasting improvement in smell loss for patients with CRSwNP, indicating the potential for comprehensive benefits beyond symptom management (46). Mullol and colleagues evaluated the effect of dupilumab on the recovery of smell in patients with CRSwNP (47). The results showed a rapid improvement in loss of smell and University of Pennsylvania Smell Identification Test (UPSIT) scores at week 24 compared with baseline, with progressive improvements throughout the study period. According to Peters and colleagues, dupilumab demonstrated a greater improvement in loss of smell after 24 weeks compared with patients treated with other biologics (48). A *post hoc* analysis by Peters and colleagues evaluated the impact of dupilumab on taste and the correlations between taste and smell in the SINUS-24 and SINUS-52 studies (49). Dupilumab significantly improved the severity of taste loss in patients treated with the drug compared to the placebo group, at weeks 24 and 52. Furthermore, at week 24, moderate associations were observed between the improvement in taste loss and the improvements in loss of smell score, 22-item Sino-Nasal Outcome Test (SNOT-22) smell/taste, and UPSIT score.

Ottaviano and colleagues conducted a study involving 147 patients with severe uncontrolled CRSwNP, treated with dupilumab as an adjunct therapy to inhaled corticosteroids for at least 1 year (50). Interestingly, dupilumab was associated with significant improvements in patients' subjective perception of olfactory impairment, as assessed by visual analogue scale for smell (VAS-smell), and nasal obstruction (VAS-NO) parameters.

The real-world DUPIREAL observation study (51) aimed to evaluate the efficacy and safety of dupilumab during the first year of treatment, by focusing on the improvement of nasal polyps score (NPS), specific symptoms, QoL, and olfactory function. Following treatment, the QoL of patients improved significantly, as demonstrated by a decrease in SNOT-22 scores compared with baseline at 9 months. The VAS-smell score was significantly reduced, while the Sniffin' Sticks score showed a significant increase over time compared with baseline, demonstrating an improvement in olfactory function.

Lastly, preliminary evidence from a real-life Italian study suggests that olfactory recovery with dupilumab treatment does not necessarily depend on polyp volume but may instead be due to the resolution of inflammation (52). In that study, which involved 53 adults with severe uncontrolled CRSwNP receiving ongoing dupilumab as add-on therapy to mometasone furoate nasal spray, improvements were observed after 6 months of treatment in SNOT-22 scores, nasal endoscopy, the VAS scale for olfactory impairment, and the Sniffin Sticks-16 items identification test (SS-I), with a significant correlation between VAS-SS-I/SNOT-22, but no correlation between NPS and SS-I or VAS.

About a third of our respondents experienced a return of olfaction within 15 days of initiating monoclonal antibody therapy. Interestingly, for four out of ten respondents, the recovery of sense of smell was sudden, and olfaction was rated as being at the highest level in terms of both quality and intensity.

These findings align with those of a multicenter, noninterventional, retrospective, observational study conducted by Barroso and colleagues (44). This study involved 206 patients with severe asthma and CRSwNP and showed a total or partial improvement in loss of smell following treatment with all monoclonal antibodies, with no significant differences observed between the groups. Certain factors, including having atopy, a greater use of short-course systemic corticosteroids, and larger polyp size, were associated with a higher likelihood of reporting an improvement in olfaction. However, it is important to note that 61% to 64% of patients reported no improvement, with no statistical differences found between the different biologic treatments.

In our survey, half of the patients reported a complete restoration of their sense of smell following biologic therapy. This full recovery highlights the potential of monoclonal antibodies in addressing sensory impairments associated with CRSwNP. A third of the patients viewed the restoration of their sense of smell as the most significant outcome of biologic therapy and reported regaining the ability to perceive smells that they had previously lost. This reconnection with lost scents can represent a substantial improvement in their sensory experience and daily life.

The significant impact of CRSwNP on individuals is well documented. Its effect on overall HRQoL has been likened to that of chronic diseases such as chronic obstructive pulmonary disease, asthma, diabetes, end-stage renal disease, Parkinson's disease, and coronary artery disease. Moreover, individuals with CRSwNP have been found to exhibit poorer physical and mental health compared with population norms (15).

Olfactory impairment can expose individuals to potential environmental dangers, such as the inability to identify expired foods or detect smoke or gas. It can also diminish their enjoyment of pleasurable experiences and interfere with the evocation of memories (15). Despite this, the impact on patients' HRQoL is often overlooked by many healthcare professionals (53).

Our survey revealed that only a small fraction of those who had not regained their sense of smell received olfactory training. Encouragingly, when this training was provided, it proved to be effective. This underscores the importance of comprehensive treatment approaches, including olfactory training, in managing CRSwNP and improving QoL.

Current guidelines for prescribing and monitoring biologic therapy in CRSwNP are primarily based on scientific literature, with variations due to differences in emphasized criteria and cutoff definitions (54). EPOS 2020 provides updated recommendations and integrated care pathways in acute rhinosinusitis and CRS (3). The 2023 European Position Paper on Rhinosinusitis suggests using biologic therapy for patients with CRSwNP who meet at least three of the following five specific criteria: (1) presence of type 2 inflammation; (2) necessity for oral glucocorticoids; (3) significant reduction in QoL; (4) substantial loss of smell; and (5) diagnosis of concurrent asthma. The Joint Task Force on Practice Parameters provides guidelines for using intranasal corticosteroids, biologics, and aspirin therapy after desensitization to manage CRSwNP (55).

The lack of standardization in international guidelines and treatment protocols for CRSwNP can lead to variable care pathways across different nations. Numerous studies have highlighted a range of unmet needs in the treatment of patients with CRSwNP, including the significant impact of the disease on social functioning and overall QoL, the limited range of treatment options leading to a substantial number of patients suffering from uncontrolled disease progression, the lack of systematic coordination and continuity in patient care, and an insufficient understanding of the disease and its effects on patients, coupled with a lack of recognition of the psychological burden associated with the disease (56, 57).

These unmet needs underscore the critical demand for the development of more efficacious and personalized treatment strategies for patients with CRSwNP. This approach mirrors those taken for other chronic conditions such as asthma, diabetes, chronic kidney disease, Parkinson's disease, and coronary artery disease. Such conditions necessitate a personalized diagnostic and care pathway, which may include regular screening, symptom monitoring, management of comorbidities, patient education, and psychological support.

### 4.1 Strengths and limitations

The design of this study offered a cost-effective and efficient approach to data collection. This design minimizes barriers to participation and enhances the response rate. Participation in the survey was voluntary, potentially introducing self-selection bias and impacting the generalizability of the results. The study relied heavily on self-reporting, and the lack of strict inclusion or exclusion criteria may make it more challenging to draw definitive conclusions from the data. The limited details of demographics and comorbidities can limit the ability to control for potential confounding variables. This could affect the interpretation of the results. Data from a country such as Italy, where food is entrenched in culture, olfaction ability affects quality of eating (12, 36, 58); therefore Italians may have been more sensitive to the QoL changes resulting from olfactory impairment. The response rate may have been affected by technical limitations and/or participants' computer and language proficiency.

## 5 Conclusions

This survey-based study of individuals living with CRSwNP provides valuable insights into the impact of olfactory impairment in this patient population. CRSwNP significantly affects patient QoL, with olfactory dysfunction being a common symptom. The treatment of CRSwNP often requires multiple and potentially indefinite therapies. However, these treatments may not always result in a long-term restoration of smell. Biologic therapy, specifically with monoclonal antibodies such as dupilumab, has shown promising results in managing CRSwNP and improving olfactory function. This treatment led to rapid and sustained improvement in smell, with about half of the patients reporting a complete restoration of their sense of smell. Despite the significant impact of olfactory impairment on patients’ HRQoL, healthcare professionals often overlook this aspect. Current guidelines for prescribing and monitoring biologic therapy in CRSwNP exhibit similarities but also differences due to variations in emphasized criteria and specific cutoff definitions. This lack of standardization can result in variable care pathways across different nations. There are several unmet needs in the treatment of patients with CRSwNP, including the significant impact of the disease on social functioning and overall QoL, the limited range of treatment options, the lack of systematic coordination and continuity in patient care, and the insufficient understanding of the disease and its effects on patients. Similar to the approach taken for other chronic conditions, these unmet needs in CRSwNP highlight the demand for the development of more efficacious and personalized treatment strategies.

## Data Availability

The raw data supporting the conclusions of this article will be made available by the authors, without undue reservation.
